# Efficacy and Safety of Non-Steroidal Mineralocorticoid Receptor Antagonists in Patients With Chronic Kidney Disease and Type 2 Diabetes: A Systematic Review Incorporating an Indirect Comparisons Meta-Analysis

**DOI:** 10.3389/fphar.2022.896947

**Published:** 2022-06-16

**Authors:** Xinrui Jiang, Zhengji Zhang, Chunlu Li, Shijin Zhang, Qiang Su, Siyun Yang, Xin Liu, Ying Hu, Xiaofeng Pu

**Affiliations:** ^1^ Nanchong Key Laboratory of Individualized Drug Therapy, Department of Pharmacy, The Second Clinical Medical College of North Sichuan Medical College, Nanchong Central Hospital, Nanchong, China; ^2^ Department of Pharmacy, The Affiliated Hospital of Southwest Medical University, Luzhou, China; ^3^ Department of Pharmacy, Chengdu Second People’s Hospital, Chengdu, China

**Keywords:** finerenone, apararenone, esaxerenone, mineralocorticoid receptor antagonists, type 2 diabetes, chronic kidney disease, meta-analysis

## Abstract

**Background:** The non-steroidal mineralocorticoid receptor antagonists (MRAs) are promising treatments in patients with chronic kidney disease (CKD) and type 2 diabetes (T2D). We conducted a meta-analysis to explore the efficacy and safety of the non-steroidal MRAs (finerenone, apararenone, esaxerenone) and detect the differences among them.

**Methods:** We searched several databases for eligible randomized controlled trials (RCTs) investigating non-steroidal MRAs versus placebo in patients with CKD and T2D. We performed a conventional meta-analysis separately, and then indirect comparisons for efficacy and safety outcomes were conducted among these included drugs.

**Results:** Eight RCTs with 14,450 subjects were enrolled. In patients with CKD and T2D, a greater reduction in urinary albumin-to-creatinine ratio (UACR) (WMD −0.40, 95% CI −0.48 to −0.32, *p* < 0.001), estimated glomerular filtration rate (eGFR) (WMD −2.69, 95% CI −4.47 to −0.91, *p* = 0.003), systolic blood pressure (SBP) (WMD −4.84, 95% CI −5.96 to −3.72, *p* < 0.001) and a higher risk of hyperkalemia (RR 2.07, 95% CI 1.86 to 2.30, *p* < 0.001) were observed in the non-steroidal MRAs versus placebo; there is no significant difference in the incidence of serious adverse events between two groups (RR 1.32, 95% CI 0.98 to 1.79, *p* = 0.067). Compared with finerenone, esaxerenone showed no significant difference in UACR reduction (WMD 0.24, 95% CI −0.016 to 0.496, *p* = 0.869); apararenone and esaxerenone showed greater decreases in SBP (WMD 1.37, 95% CI 0.456 to 2.284, *p* = 0.010; WMD 3.11, 95% CI 0.544 to 5,676, *p* = 0.021).

**Conclusions:** Despite the moderate increased risk of hyperkalemia, use of non-steroidal MRAs could reduce proteinuria and SBP in patients with CKD and T2D. In terms of renoprotection, esaxerenone and finerenone may have similar effects. Esaxerenone and apararenone may have better antihypertensive effects than finerenone. The head-to-head RCTs are still needed to compare the differences of the efficacy and safety in these non-steroidal MRAs.

## Introduction

Type 2 diabetes (T2D) is a vital public health problem because of its increasing burden in daily life. The kidney is evidently the most important target of microvascular damage in diabetes (Thomas et al., 2015). Currently, T2D has become the leading cause of chronic kidney disease (CKD) worldwide (Li et al., 2021). It is estimated that approximately 40% of patients with T2D will develop CKD (Thomas et al., 2015). T2D with CKD is usually associated with increased serum creatinine, persistent albuminuria, and gradually decreased estimated glomerular filtration rate (eGFR) (Norris et al., 2018). If CKD in patients with T2D is not adequately treated, it can lead to end-stage renal disease (ESRD) (Thomas et al., 2015).

Currently, therapeutic principles for patients with CKD and T2D are mainly control of hypertension and hyperglycemia; renin-angiotensin system (RAS) blockers (angiotensin-receptor blockers [ARBs] or angiotensin-converting-enzyme [ACE] inhibitors), sodium-glucose cotransporter 2 (SGLT2) inhibitors, and glucagon-like peptide-1 receptor agonists (GLP-1RAs) are all considered effective treatment strategies (Delanaye and Scheen, 2019; American Diabetes Association, 2020). Although these therapies have benefits for patients with CKD and T2D, a risk of CKD progression still remains (Perkovic et al., 2019). Aldosterone, a mineralocorticoid hormone, is a downstream target of the activation of the renin-angiotensin-aldosterone system (RAAS), which may influence human kidneys by increasing proteinuria and decreasing renal function (Ingelfinger and Rosen, 2020). Activation of the aldosterone system in the diabetic kidney result in inflammation, proliferation, fibrosis, and ESRD. Besides, high circulating levels of aldosterone are also related to insulin resistance and endothelial dysfunction, which further aggravate the progression of CKD in patients with T2D (Kang and Cha, 2009). Therefore, blockading the mineralocorticoid receptor (MR) might be an available strategy to treat CKD in patients with T2D. Several studies have shown that classic steroidal mineralocorticoid receptor antagonists (MRAs) (spironolactone and eplerenone) have moderate protective effects on the kidney (Bianchi et al., 2006; Epstein et al., 2006; Sun et al., 2017; Weir et al., 2018). However, hyperkalemia and other adverse effects impede the routine use of classic steroidal MRAs (Al Dhaybi and Bakris, 2020). Therefore, new therapies are needed for patients with CKD and T2D.

Finerenone, apararenone, and esaxerenone are novel non-steroidal MRAs that provide less risk for hyperkalemia, with similar benefits of aldosterone blockade (Al Dhaybi and Bakris, 2020). Some preclinical studies indicate that finerenone and esaxerenone have a high potency and selectivity for MR compared with spironolactone and eplerenone (Bärfacker et al., 2012; Takahashi et al., 2020). Besides, it was reported that finerenone had greater renoprotection and a lower risk of hyperkalemia than spironolactone and eplerenone (Pitt et al., 2013; Kolkhof et al., 2014). Simultaneously, we are curious about the effects of apararenone and esaxerenone on patients with CKD and T2D. Recently, several clinical studies about apararenone and esaxerenone have revealed their trial results (European Union Clinical Trials Register, 2016; Ito et al., 2019; Ito et al., 2020; Wada et al., 2021). However, there are no meta-analyses exploring the efficacy and safety of apararenone and esaxerenone in patients with CKD and T2D. Moreover, no head-to-head randomized controlled trials (RCTs) have investigated the differences in the efficacy and safety of finerenone, apararenone, and esaxerenone in patients with CKD and T2D. Thus, we decided to conduct a meta-analysis to explore the efficacy and safety of non-steroidal MRAs (finerenone, apararenone, and esaxerenone) in patients with CKD and T2D; besides, an indirect treatment–comparison technique was used to explore which drug might be superior in the treatment of CKD in patients with type 2 diabetes.

## Methods

### Protocol and Guidance

The study protocol was registered in the International Database of Prospectively Registered Systematic Reviews (PROSPERO; Registration No. CRD42021272482) https://www.crd.york.ac.uk/PROSPERO/display_record.php?RecordID=272482. We followed the Preferred Reporting Items for Systematic reviews and Meta-Analyses (PRISMA) reporting guideline (Moher et al., 2009).

### Data Sources and Searches

We searched Medline (PubMed), Embase, the Cochrane Central Register of Controlled Trials (CENTRAL), and ClinicalTrials.gov from inception to 5 December 2021. We also searched PROSPERO for any ongoing or recently completed systematic reviews. The geographic area and language were not restricted. Supplementary Table S1 presents the detailed search strategy.

### Inclusion Criteria

We considered trials eligible if they enrolled adults (age ≥ 18 years) who were diagnosed with T2D and CKD. The intervention of the included studies should be non-steroidal MRAs, and the comparison should be placebo. Furthermore, the trials were considered to be eligible if the they contained at least one of the following outcomes: changes in urinary albumin-to-creatinine ratio (UACR) from baseline, changes in eGFR from baseline, changes in systolic blood pressure (SBP) from baseline, incidence of hyperkalemia, serious adverse events (SAEs), a sustained decrease of 40% in the eGFR from baseline, kidney failure, death from cardiovascular causes, nonfatal myocardial infarction, nonfatal stroke, and hospitalization for heart failure. The type of study should be RCT.

### Exclusion Criteria

We excluded studies if they were non-RCT studies (e.g., case reports or observational studies); if the patients were complicated with type 1 diabetes; and if studies were published in reviews, abstracts, or protocols.

### Assessment of Risk of Bias in Included Studies

Two reviewers (XRJ and XFP) assessed the quality of each included study according to the Cochrane Handbook for Systematic Reviews of Interventions (Cumpston et al., 2019). We assessed the risk of bias according to the following items: random sequence generation, allocation concealment, blinding of participants and investigators, blinding of outcome assessment, incomplete outcome data, selective outcome reporting, and other bias (e.g., funding source). The risk of bias was assessed by two reviewers independently, and disagreements were resolved by discussion and consensus.

### Outcomes

The primary outcome was changes in UACR from baseline. Secondary outcomes were changes in eGFR from baseline, changes in SBP from baseline, incidence of hyperkalemia, a sustained decrease of 40% in the eGFR from baseline, kidney failure, death from cardiovascular causes, nonfatal myocardial infarction, nonfatal stroke, and hospitalization for heart failure and SAEs.

### Data Extraction

The following study data were extracted by two reviewers (XRJ and ZJZ) from the included studies: trial details (e.g., authors, year of publication, region); subjects (e.g., sample size, mean age, gender, eGFR at baseline, UACR at baseline, and SBP at baseline); interventions (e.g., treatment regimen, duration); outcomes (e.g., changes in UACR from baseline, changes in eGFR from baseline, incidence of hyperkalemia and SAEs, etc.). The data were extracted from original articles and online clinical trial websites and checked for accuracy by two reviewers (XRJ and XFP).

### Data Analysis

Herein, Stata software version 16.0 (StataCorp, TX, United States ) was used to perform statistical analysis. We used weighted mean differences (WMDs) and their associated 95% confidence intervals (CIs) to assess continuous outcomes (e.g., changes in UACR from baseline and changes in eGFR from baseline, etc.). Besides, we used risk ratios (RRs) and their associated 95% CIs to assess dichotomous outcomes (e.g., incidence of hyperkalemia and SAEs, etc.). Statistical heterogeneity was assessed using the I^2^ test. If significant heterogeneity was not present (I^2^< 50%), we used fixed effects models to pool outcomes; we used random effects models when significant heterogeneity was present (I^2^ ≥ 50%). We preliminarily assessed the publication bias by funnel plot, and then the Egger test and Begg test were used to do further analyses.

Sensitivity analyses were performed by removing one study at a time to explore whether the heterogeneity was significantly reduced and further matching baseline characteristics to detect the stability of the results of direct and indirect comparisons. Indirect treatment comparison was performed according to Bucher et al. (1997). We used ITC (Indirect Treatment Comparison, Version 1.0, Ottawa: Canadian Agency for Drugs and Technologies in Health) software to detect the differences of efficacy and safety between the included drugs (finerenone, apararenone, and esaxerenone). This indirect comparison was made through a common comparator (placebo group). The efficacy and safety of included drugs was considered significantly different if *p* < 0.05 and the 95% CI did not contain a WMD = 0 or an RR = 1.

## Results

### Eligible Studies and Study Characteristics

**FIGURE 1:**
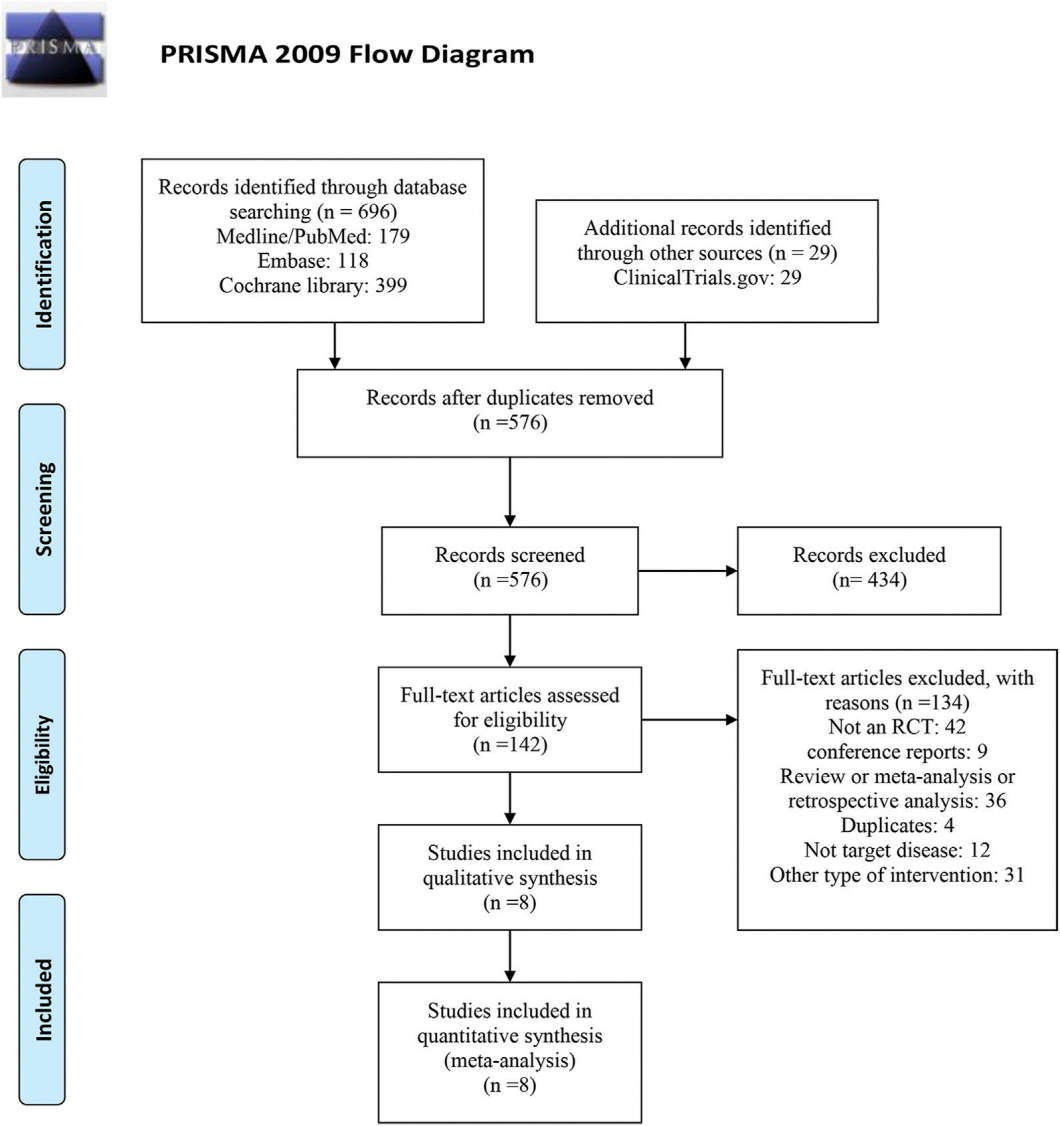
PRISMA flow diagram for study selection.

As shown in Figure1, of the 725 studies we retrieved from the aforementioned databases and other sources, we identified eight RCTs (Bakris et al., 2015; European Union Clinical Trials Register, 2016; Katayama et al., 2017; Ito et al., 2019; Bakris et al., 2020; Ito et al., 2020; Pitt et al., 2021; Wada et al., 2021) with 14,450 subjects that met the inclusion criteria. Detailed characteristics included in RCTs are presented in Table 1. The years of publication of these RCTs ranged from 2015 to 2021. Four trials were performed in multiple countries, and the other four trials were performed in Japan. Among the eight enrolled studies, four studies investigated finerenone, two studies investigated apararenone, and the other two studies investigated esaxerenone. For the included dose regimens, the finerenone group selected 10 and 20 mg/d; the esaxerenone group selected 1.25, 2.5, and 5 mg/d; and the apararenone group selected 2.5, 5, and 10 mg/d. The selections of these dose regimens were according to previous studies (Al Dhaybi and Bakris, 2020; Fu et al., 2021). For each outcome in each drug, these dose groups were combined into one group, which showed the pooled results.

**TABLE 1:** Characteristics of included studies.

Source	Region	Treatment	No. of patients (M/F)	Mean age, years	Systolic BP, mm Hg	eGFR at baseline (mL/min/1.73 m^2^)	UACR at baseline (mg/g)	Duration, weeks
Bakris et al. (2015)	23 countries	Finerenone:10 mg/d, 20 mg/d	F-10 mg: 98 (77/21)	F-10 mg: 64.94 ± 9.62^*^	F-10 mg: 137.6 ± 14.0^*^	F-10 mg: 67.0 ± 20.9^*^	F-10 mg: 230.7 ± 171.0^*^	12
			F-20 mg: 119 (89/30)	F-20 mg: 64.70 ± 9.26^*^	F-20 mg: 138.1 ± 14.3^*^	F-20 mg: 66.0 ± 22.2^*^	F-20 mg: 204.1 ± 171.9^*^	
			C: 94 (69/25)	C: 63.26 ± 8.68^*^	C:139.9 ± 14.3^*^	C: 72.2 ± 20.4^*^	C: 188.4 ± 169.8^*^	
Katayama et al. (2017)	Japan	Finerenone:10 mg/d, 20 mg/d	F-10 mg: 12	F-10 mg: 62.75 ± 7.06^*^	F-10 mg: 140.33 ± 14.84^*^	F-10 mg: 69.79 ± 12.17^*^	F-10 mg: 203.19 ± 464.84^*^	12
			F-20 mg: 12	F-20 mg: 64.00 ± 8.26^*^	F-20 mg: 141.72 ± 18.39^*^	F-20 mg: 61.48 ± 11.01^*^	F-20 mg: 150.43 ± 97.68^*^	
			C: 12	C: 66.75 ± 9.02^*^	C: 135.72 ± 16.90^*^	C: 60.88 ± 16.53	C: 256.80 ± 166.31^*^	
Bakris et al. (2020)	48 countries	Finerenone:10–20 mg/d	F: 2833 (1953/880)	F: 65.4 ± 8.9^*^	F: 138.1 ± 14.3^*^	F: 44.4 ± 12.5^*^	F: 833 (441–1628)^#^	125
			C: 2841 (2030/811)	C: 65.7 ± 9.2^*^	C: 138.0 ± 14.4^*^	C: 44.3 ± 12.6^*^	C: 867 (453–1645)^#^	
Pitt et al. (2021)	48 countries	Finerenone: 10–20 mg/d	F: 3686 (2528/1158)	F: 64.1 ± 9.7^*^	F: 135.8 ± 14.0^*^	F: 67.6 ± 21.7^*^	F:302 (105–749) ^#^	163
			C: 3666 (2577/1089)	C: 64.1 ± 10.0^*^	C: 135.7 ± 14.1^*^	C: 68.0 ± 21.7^*^	C:315 (111–731) ^#^	
NCT01756716 European Union Clinical Trials Register (2016)	6 countries	Apararenone: 2.5 mg/d, 5 mg/d	A-2.5 mg: 17 (12/5) A-5 mg: 16 (10/6) C: 16 (11/5)	A-2.5 mg: 62.1 ± 7.1^*^ A-5 mg: 62.1 ± 7.1^*^ C: 66.4 ± 4.5^*^	NA	NA	NA	8
Wada et al. (2021)	Japan	Apararenone: 2.5 mg/d, 5 mg/d, 10 mg/d	A-2.5 mg: 73 (52/21)	A-2.5 mg: 63.2 ± 8.5^*^	A-2.5 mg: 136.6 ± 11.8^*^	A-2.5 mg: 70.9 ± 17.9^*^	A-2.5 mg:151.18 ± 88.37^*^	24
			A-5 mg: 74 (57/17)	A-5 mg: 61.7 ± 9.0^*^	A-5 mg:136.0 ± 11.6^*^ A-10mg: 135.0 ± 12.5^*^	A-5 mg: 78.3 ± 20.4^*^	A-5 mg: 131.91 ± 87.54^*^	
			A-10 mg: 73 (58/15)	A-10 mg:62.1 ± 9.5^*^	C: 133.4 ± 11.8^*^	A-10 mg: 73.0 ± 21.8^*^	A-10 mg:130.20 ± 68.34^*^	
			C: 72 (54/18)	C:60.1 ± 10.0^*^		C: 77.5 ± 20.5^*^	C:141.62 ± 88.23^*^	

Source	Region	Treatment	No. of patients (M/F)	Mean age, years	Systolic BP, mm Hg	eGFR at baseline (mL/min/1.73 m^2^)	UACR at baseline (mg/g)	Duration, weeks
Ito et al. (2019)	Japan	Esaxerenone: 1.25 mg/d, 2.5 mg/d, 5 mg/d	E-1.25 mg:72(54/18)	E-1.25 mg: 66 ± 8^*^	E-1.25 mg: 139 ± 10^*^	E-1.25 mg: 66 ± 18^*^	E-1.25 mg: 111 ± 58.45^*^	12
			E-2.5 mg: 70 (57/13)	E-2.5 mg: 64 ± 11^*^	E-2.5 mg: 137 ± 13^*^	E-2.5 mg: 68 ± 19^*^	E-2.5 mg: 112 ± 55.50^*^	
			E-5 mg: 72 (57/15)	E-5mg: 65 ± 9^*^	E-5mg:136 ± 12^*^C: 138 ± 11^*^	E-5mg: 69 ± 18^*^	E-5 mg: 110 ± 54.12^*^	
			C: 73 (57/16)	C: 66 ± 10^*^		C: 69 ± 19^*^	C: 110 ± 54.50^*^	
Ito et al. (2020)	Japan	Esaxerenone: 1.25–2.5 mg/d	E- 1.25–2.5 mg/d	E-1.25–2.5 mg/d	E-1.25–2.5 mg/d	E-1.25–2.5 mg/d: 69 ± 18^*^	E-1.25–2.5 mg/d	52
			222 (165/57)	66 ± 10^*^	140 ± 10^*^	C: 69 ± 18^*^	113 (46, 286)^$^	
			C: 227 (180/47)	C: 66 ± 9^*^	C: 140 ± 10^*^		C:110(47, 278)^$^	

A, apararenone; BP, blood pressure; C, control group; E, esaxerenone; eGFR, estimated glomerular filtration rate; F, finerenone; d, day; NA, not available; UACR, urinary albumin-creatinine ratio; *Mean ± SD; #Median (interquartile range); $Median (min, max).

### Risk of Bias in Included Studies

**FIGURE 2:**
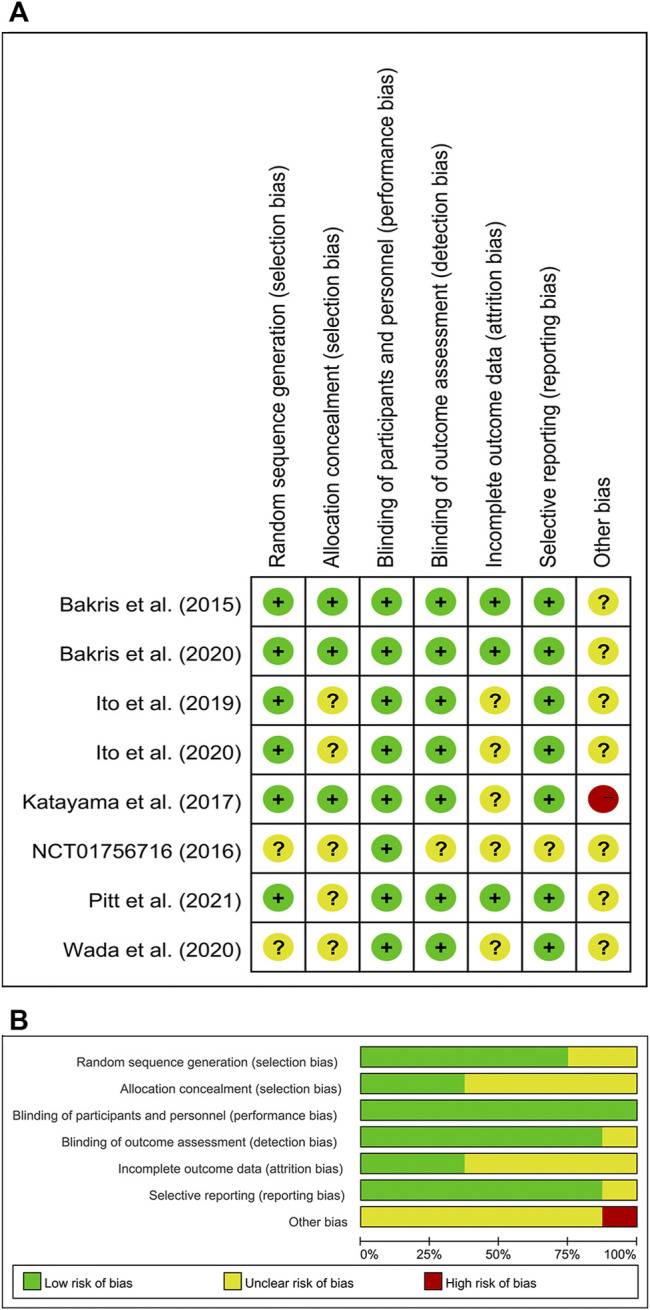
**(A)** Risk of bias summary for included studies, showing each risk of bias item for every included study. **(B)** Risk of bias graph presenting each risk of bias item as percentages across all included studies.

Figure 2 showed the risk of bias of the included studies. Six studies were considered low risk for random sequence generation, which was unclear in the other two studies due to a lack of information. Three studies were considered low risk for allocation concealment, which was unclear in the other five studies. All eight studies were considered low risk for blinding of participants and personnel. Seven studies were considered low risk for blinding of outcome assessment, and one study was considered unclear risk for blinding of outcome assessment. Three studies were considered low risk for incomplete outcome data, which was unclear in the other five studies. Seven studies were considered low risk for incomplete outcome data, which was unclear in one study. Other source of bias was unclear in seven studies because they were all funded by the pharmaceutical manufacturers, and the other one study was considered high risk owing to small sample size.

### Primary Outcomes

**FIGURE 3:**
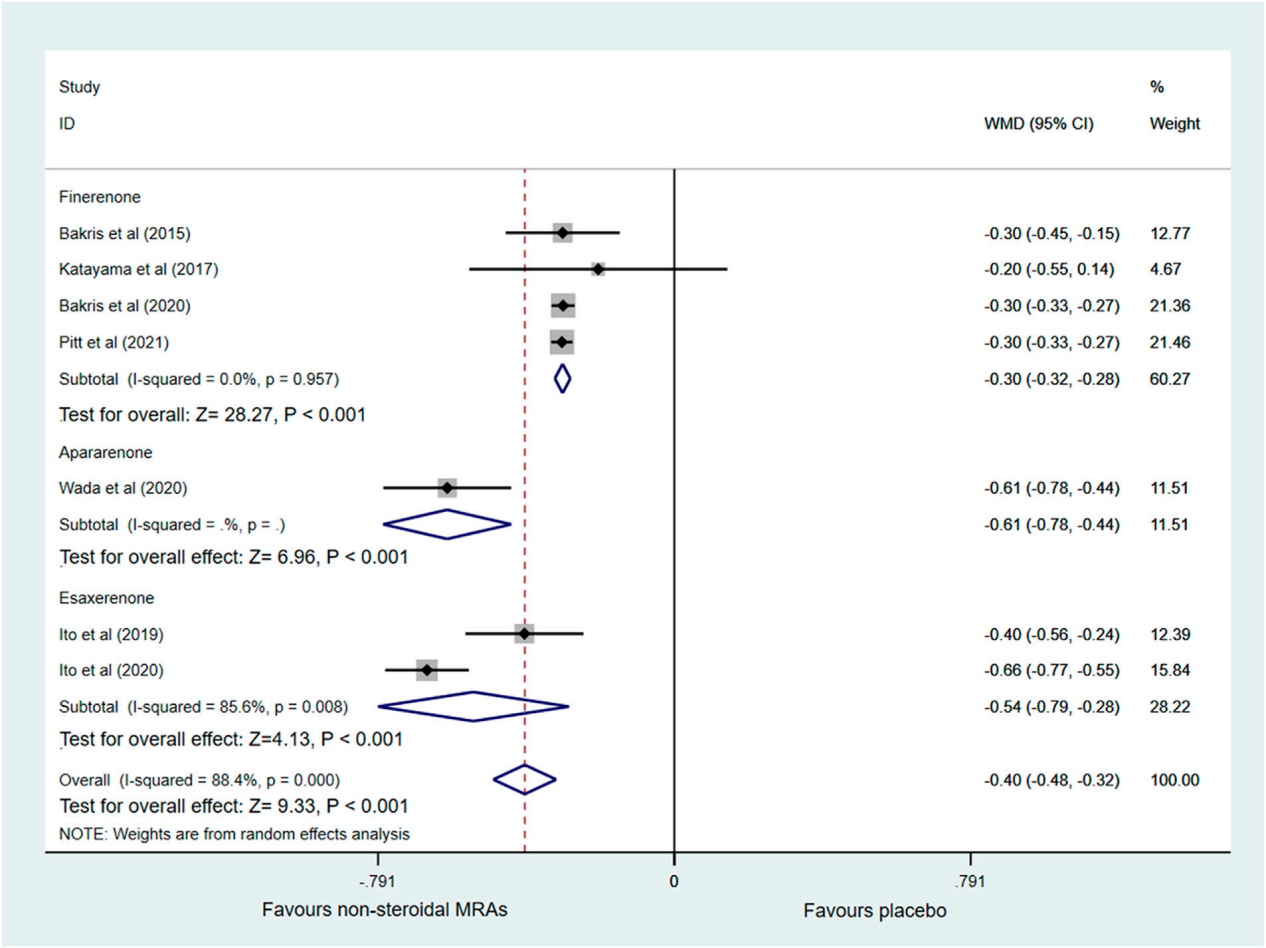
Forest plot for the effect of non-steroidal MRAs on the changes in UACR from baseline in patients with CKD and T2D. UACR, urinary albumin-to-creatinine ratio (expressed in altered ratio); WMD, weighted mean difference; MRAs, mineralocorticoid receptor antagonists; CKD, chronic kidney disease; T2D, type 2 diabetes.

Changes in UACR from baseline were reported in seven out of eight RCTs (Bakris et al., 2015; Katayama et al., 2017; Ito et al., 2019; Bakris et al., 2020; Ito et al., 2020; Pitt et al., 2021; Wada et al., 2021) with 14,401 patients, as shown in Figure 3. A significantly greater reduction in UACR among patients with CKD and T2D was observed in the non-steroidal MRAs group vs. placebo group (WMD −0.40, 95% CI −0.48 to −0.32, *p* < 0.001). The finerenone, apararenone, and esaxerenone groups all showed a significant reduction compared with the placebo group (WMD −0.30, 95% CI −0.32 to −0.28, *p* < 0.001; WMD −0.61, 95% CI −0.78 to −0.44, *p* < 0.001; WMD −0.54, 95% CI −0.79 to −0.28, *p* < 0.001). Obvious statistical heterogeneity (I^2^ = 85.6%) was found in the esaxerenone subgroup, which might result from the different treatment durations (12 vs. 52 weeks). Supplementary Figure S1 showed that the funnel plot was roughly symmetrical. In addition, the Begg test (*p* = 0.548) and Egger test (*p* = 0.181) detected no significant publication bias.

### Secondary Outcomes

**FIGURE 4:**
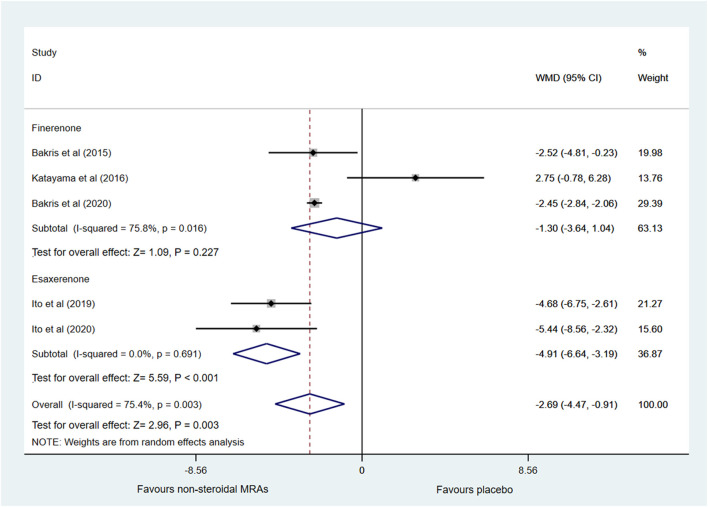
Forest plot for the effect of non-steroidal MRAs on the changes in eGFR from baseline in patients with CKD and T2D. eGFR, estimated glomerular filtration rate (expressed in mL/min/1.73 m^2^); WMD, weighted mean difference; MRAs, mineralocorticoid receptor antagonists; CKD, chronic kidney disease; T2D, type 2 diabetes.

Changes in eGFR from baseline were reported in five RCTs (Bakris et al., 2015; Katayama et al., 2017; Ito et al., 2019; Bakris et al., 2020; Ito et al., 2020), which included finerenone and esaxerenone, as shown in Figure 4. Compared with the placebo group, the non-steroidal MRAs group showed a greater reduction in eGFR in patients with CKD and T2D (WMD -2.69, 95% CI -4.47 to -0.91, *p* < 0.003). Besides, subgroup analysis indicated that compared with the placebo group, the esaxerenone group showed a greater reduction in eGFR (WMD −4.91, 95% CI −6.64 to −3.19, *p* < 0.001), but the finerenone group did not show a difference in eGFR changes (WMD −1.30, 95% CI −3.64 to 1.04, *p* = 0.227). Significant statistical heterogeneity (I^2^ = 75.4%) was found in the pooled effect estimate, and the finerenone subgroup also showed significant statistical heterogeneity (I^2^ = 75.8%). The heterogeneity (I^2^) was reduced to 0% when one study with small sample size (Katayama et al., 2017) was removed in the finerenone subgroup, and heterogeneity (I^2^) was decreased to 60.7% in the total pooled effect estimate accordingly. After removing that study, the pooled WMD of the finerenone subgroup was altered to −2.45 (95% CI −2.83 to −2.07, *p* < 0.001), and the pooled WMD of non-steroidal MRAs vs. placebo was altered to −3.40 (95% CI −4.82 to −1.97, *p* < 0.001).

Three RCTs (Bakris et al., 2015; Bakris et al., 2020; Pitt et al., 2021) investigated the incidence of a sustained decrease of 40% in the eGFR from baseline which only included finerenone, as shown in Supplementary Figure S2A. The incidence of a sustained decrease of 40% in the eGFR from baseline among patients with CKD and T2D was lower in the non-steroidal MRA (i.e., finerenone) group vs. the placebo group (RR 0.85, 95% CI 0.78 to 0.92, *p* < 0.001). There was no significant statistical heterogeneity (I^2^ = 0.0%) in the pooled effect estimate.

**FIGURE 5:**
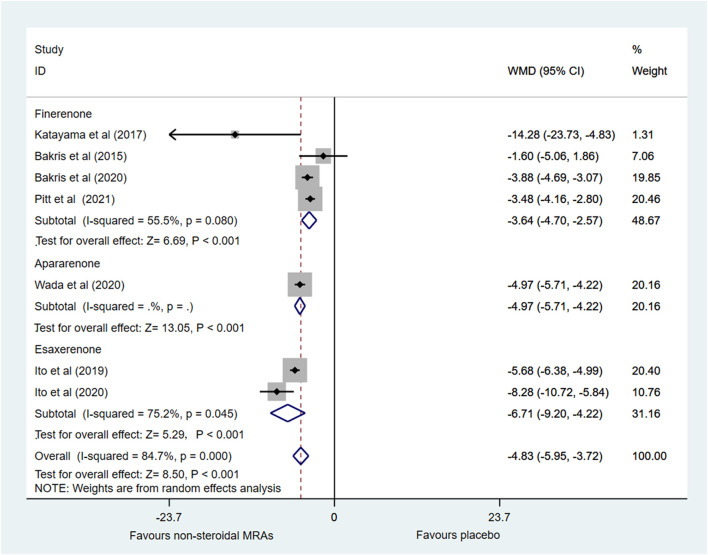
Forest plot for the effect of non-steroidal MRAs on the changes in SBP from baseline in patients with CKD and T2D. SBP, systolic blood pressure (expressed in mmHg); WMD, weighted mean difference; MRAs, mineralocorticoid receptor antagonists; CKD, chronic kidney disease; T2D, type 2 diabetes.

Changes in SBP from baseline was investigated in seven RCTs (Bakris et al., 2015; Katayama et al., 2017; Ito et al., 2019; Bakris et al., 2020; Ito et al., 2020; Pitt et al., 2021; Wada et al., 2021). Figure 5 showed that compared with the placebo group, the non-steroidal MRA group had lower SBP in patients with CKD and T2D (WMD −4.83, 95% CI −5.95 to −3.72, *p* < 0.001). Subgroup analysis indicated that compared with the placebo group, the finerenone, apararenone, and esaxerenone groups all significantly decreased SBP in patients with CKD and T2D (WMD −3.64, 95% CI −4.73 to −2.54, *p* < 0.001; WMD −4.97, 95% CI −5.71 to −4.22, *p* < 0.001; WMD −6.71, 95% CI −9.20 to −4.22, *p* < 0.001). However, there was significant statistical heterogeneity (I^2^ = 84.7%) in the pooled effect estimates, and the finerenone and esaxerenone subgroups both showed marked heterogeneity (I^2^ = 56.8% and 75.2%). There were only two studies in the esaxerenone subgroup, so we could not conduct the sensitivity analysis by removing one study. After we removed one small sample size study (Katayama et al., 2017), the heterogeneity (I^2^) of the finerenone subgroup was decreased to 2.7%, and the pooled effect estimate of the finerenone subgroup remained almost unchanged. However, the total statistical heterogeneity remained nearly unchanged.

**FIGURE 6:**
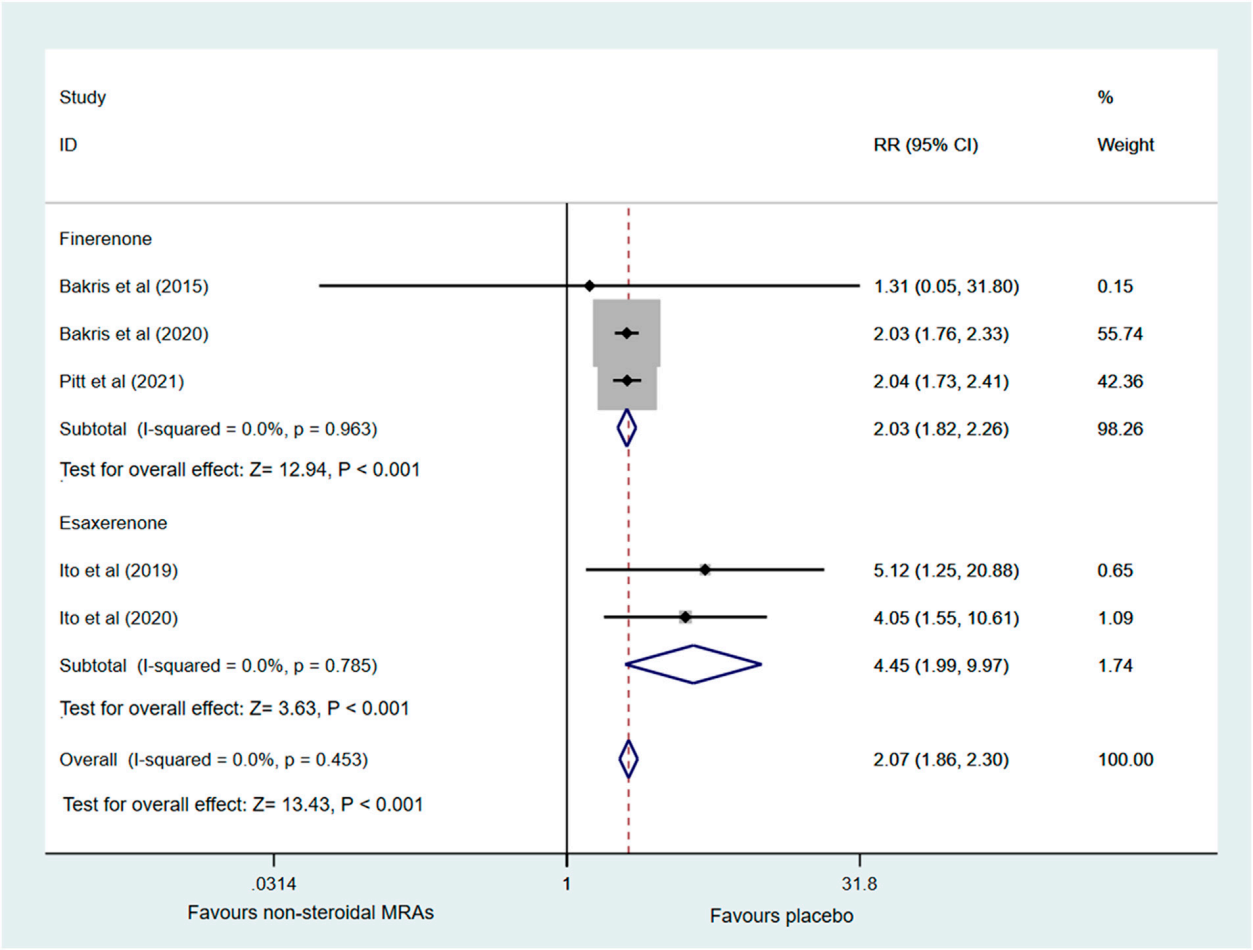
Forest plot for the effect of non-steroidal MRAs on the incidence of hyperkalemia in patients with CKD and T2D. RR, risk ratio; MRAs, mineralocorticoid receptor antagonists; CKD, chronic kidney disease; T2D, type 2 diabetes.

Five RCTs (Bakris et al., 2015; Ito et al., 2019; Bakris et al., 2020; Ito et al., 2020; Pitt et al., 2021) reported the incidence of hyperkalemia involving finerenone and esaxerenone, as shown in Figure 6. A higher incidence of hyperkalemia among patients with CKD and T2D was found in the non-steroidal MRA group vs. placebo group (RR 2.07, 95% CI 1.86 to 2.30, *p* < 0.001). There was no significant statistical heterogeneity (I^2^ = 0.0%) in the pooled effect estimate. Subgroup analysis showed that finerenone and esaxerenone both led to a higher risk of hyperkalemia (RR 2.03, 95% CI 1.82 to 2.26, *p* < 0.001; RR 4.45, 95% CI 1.99 to 9.97, *p* < 0.001).

**FIGURE 7:**
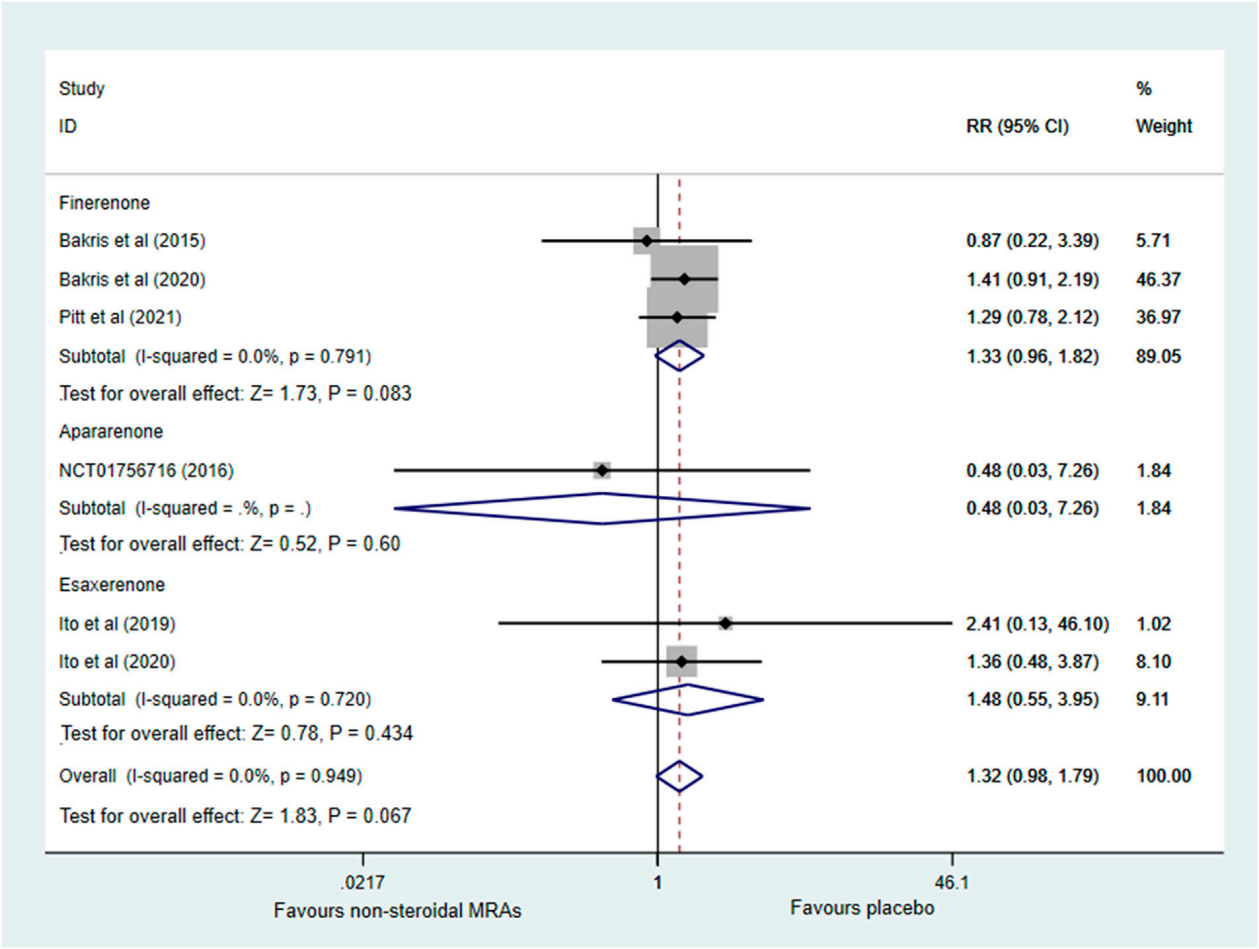
Forest plot for the effect of non-steroidal MRAs on the incidence of serious adverse events in patients with CKD and T2D. RR, risk ratio; MRAs, mineralocorticoid receptor antagonists; CKD, chronic kidney disease; T2D, type 2 diabetes.

Six RCTs (Bakris et al., 2015; European Union Clinical Trials Register, 2016; Ito et al., 2019; Bakris et al., 2020; Ito et al., 2020; Pitt et al., 2021) investigated the incidence of serious adverse events. Figure 7 indicated that no significant difference was noted between the non-steroidal MRA group and the placebo group among patients with CKD and T2D (RR 1.32, 95% CI 0.98 to 1.79, *p* = 0.067), and the pooled effect estimate showed no obvious statistical heterogeneity (I^2^ = 0.0%). Subgroup analysis showed that the finerenone, apararenone, and esaxerenone groups did not significantly differ from the placebo group (RR 1.33, 95% CI 0.96 to 1.82, *p* = 0.083; RR 0.48, 95% CI 0.03 to 7.26, *p* = 0.600; RR 1.48, 95% CI 0.55 to 3.95, *p* = 0.434).

The outcomes of kidney failure, death from cardiovascular causes, nonfatal myocardial infarction, nonfatal stroke, and hospitalization for heart failure were only involved in the finerenone group (detailed results showed in Supplementary Figure S2).

The Supplementary Table S2 showed the results of direct and indirect comparisons of included studies after matching baseline UACR and eGFR. The results of changes in UACR from baseline and incidence of hyperkalemia remained almost unchanged. However, changes in eGFR from baseline became not significant different between non-steroidal MRAs group and placebo group (WMD −2.64, 95% CI −5.65 to 0.36), *p* = 0.084). Meanwhile, the results of indirect comparisons of changes in eGFR from baseline showed the similar changes between finerenone group and esaxerenone group (WMD 4.85, 95% CI −1.669 to 11.369, *p* = 0.083).

### Indirect Treatment Comparison

Table 2 shows the results of indirect treatment comparison. For the outcome of changes in UACR from baseline, the apararenone group showed greater reduction compared with the finerenone group in patients with CKD and T2D (WMD 0.31, 95% CI 0.139 to 0.481, *p* = 0.004). No significant difference was noted between the finerenone group and the esaxerenone group in changes in UACR (WMD 0.24, 95% CI −0.016 to 0.496, *p* = 0.869). Similarly, there was no significant difference between apararenone and esaxerenone in changes in UACR (WMD −0.07, 95% CI −0.376 to 0.236, *p* = 0.979). For the outcome of changes in eGFR from baseline, the esaxerenone group showed a greater reduction compared with finerenone group (WMD 2.46, 95% CI 0.694 to 4.226, *p* = 0.041). The incidence of hyperkalemia of the finerenone group did not significantly differ from that of the esaxerenone group (RR 0.456, 95% CI 0.202 to 1.028, *p* = 0.138). For the outcome of changes in SBP from baseline, compared with the finerenone group, apararenone and esaxerenone showed superiority in the decline of SBP levels (WMD 1.37, 95% CI 0.456 to 2.284, *p* = 0.010; WMD 3.11, 95% CI 0.544 to 5,676, *p* = 0.021). Besides, no significant difference in SBP changes from baseline was noted between apararenone and esaxerenone (WMD 1.74, 95% CI −0.859 to 4.339, *p* = 0.115).

**TABLE 2:** Indirect comparisons between finerenone, apararenone, and esaxerenone in patients with T2D and CKD.

Outcomes	Comparisons	Effect estimates (95% CI)^a^	*p* Value
Changes in UACR from baseline	Finerenone vs. Apararenone	0.31 (0.139–0.481)	0.004
	Finerenone vs. Esaxerenone	0.24 (−0.016–0.496)	0.869
	Apararenone vs. Esaxerenone	−0.07 (−0.376–0.236)	0.979
Changes in eGFR from baseline	Finerenone vs. Esaxerenone	2.46 (0.694–4.226)	0.041
Incidence of hyperkalemia	Finerenone vs. Esaxerenone	0.456 (0.202–1.028)	0.138
Changes in SBP from baseline	Finerenone vs. Apararenone	1.37 (0.461–2.279)	0.010
	Finerenone vs. Esaxerenone	3.11 (0.566–5.654)	0.021
	Apararenone vs. Esaxerenone	1.74 (−0.859–4.339)	0.115

a Effect estimates: risk ratio for incidence of hyperkalemia; weighted mean difference for changes in UACR, from baseline (expressed in altered ratio), changes in eGFR, from baseline (expressed in mL/min/1.73 m^2^), changes in SBP, from baseline (expressed in mmHg). UACR, urinary albumin-to-creatinine ratio; eGFR, estimated glomerular filtration rate; SBP, systolic blood pressure; T2D, type 2 diabetes; CKD, chronic kidney disease.

## Discussion

The main findings of this meta-analysis are that the use of non-steroidal MRAs (finerenone, apararenone, and esaxerenone) in patients with CKD and T2D decreases UACR and SBP without an excess risk of serious adverse events; however, the use of non-steroidal MRAs (finerenone and esaxerenone) in patients with CKD and T2D may lead to decreased eGFR and an increased risk of hyperkalemia. Finerenone may have benefits in reducing the incidence of a sustained decrease of 40% in the eGFR from baseline and reducing the risk of hospitalization for heart failure. In addition, the results of indirect treatment comparisons indicate that (I) apararenone is likely to be superior than finerenone in decreasing UACR among patients with CKD and T2D; (II) finerenone seems to be superior to esaxerenone in alleviating the decline in eGFR; and (III) esaxerenone and apararenone may have superiority in decreasing SBP compared with finerenone. Nonetheless, the findings of indirect treatment comparisons included the apararenone group should be interpreted with caution because there was only one RCT involved in it.

Chronic kidney disease is usually associated with persistent albuminuria, and a decrease in albuminuria is considered to have renoprotection (Lin et al., 2018). The albuminuria excretion rate, calculated as the urinary albumin-to-creatinine ratio (UACR), is used clinically to evaluate albuminuria (Gansevoort et al., 2005). The results of this meta-analysis found that finerenone, apararenone, and esaxerenone all can decrease the UACR in patients with CKD and T2D. This finding about finerenone is in line with a previous meta-analysis (Fu et al., 2021). However, the esaxerenone subgroup showed significant statistical heterogeneity (I^2^ = 85.6%), which may result from the different treatment durations (12 vs. 52 weeks). The study of Wada et al. (2021) showed that the UACR in the esaxerenone group gradually decreased over time, and approximately remained stable after 24 weeks. For the comparison of steroidal MRAs and non-steroidal MRAs in patients with CKD and T2D, there was a lack of studies or difficult to extract necessary data. Therefore, we merely could do some descriptive analyses. Several studies (Epstein et al., 2006; El Mokadem et al., 2020; Brandt-Jacobsen et al., 2021) showed that for the treatment of steroidal MRA eplerenone, the reduction of UACR was approximately 30%–40% versus placbo in patients with CKD and T2D, and the results of this meta-anlysis indicated that use of non-steroidal MRAs led to a 40% decrease in UACR versus placbo. In addition, the reduction was 61% and 54% in apararenone and esaxerenone group respectively. Thus, it seems that apararenone and esaxerenone have greater UACR reduction compared with eplerenone, but this could not reach any definitive conclusions due to a lack of statistical evidence. Control of hypertension plays an important role in the management of CKD in patients with type 2 diabetes (Delanaye and Scheen, 2019). A cohort study showed that in patients with CKD, higher blood pressure levels were associated with a higher risk of a composite kidney outcome reflecting CKD progression (Lee et al., 2021). This meta-analysis reveals that finerenone, apararenone, and esaxerenone all can decline SBP in patients with CKD and T2D; esaxerenone and apararenone may have superiority in decreasing SBP compared with finerenone. A newer non-steroidal MRA KBP 5074 (not included in this analysis) was investigated in a phase 2b study which enrolled the patients with uncontrolled or resistant hypertension and stage 3b/4 CKD (Bakris et al., 2021), and the results of this study showed that compared with the placebo, the changes in SBP from baseline was about 7–10 mmHg with the dose of KBP 5074 increased from 0.25 to 0.5 mg in these patients. The pooled results of this meta-analysis indicated that the changes in SBP from baseline (vs. placebo) was 3.64, 4.97, and 6.71 mmHg in finerenone, apararenone, and esaxerenone groups respectively. It was likely that the KBP 5074 was superior to finerenone, apararenone, and esaxerenone in the decline of SBP; nevertheless, the baseline SBP was higher in the KBP 5074 study, and it could not reach definitive conclusions due to a lack of statistical evidence. The antihypertensive effect of non-steroidal MRAs may be inferior to ARB or ACEI, but the non-steroidal MRAs do decrease the SBP levels versus placebo in this work. Apararenone (MT-3995), discovered by Mitsubishi Tanabe Pharma Corporation (Tokyo, Japan), is a non-steroidal compound with highly selective MRA activity. Wada et al. (2021) reported that apararenone may have superior efficacy than similar drugs via data from nonclinical studies. Our results of indirect treatment comparisons showed that apararenone might be superior to finerenone in decreasing UACR and SBP among patients with CKD and T2D. Thus, this finding further confirms the superiority of apararenone in treating patients with CKD and T2D. In addition, we found that esaxerenone and finerenone were likely to have similar effects in UACR reduction. Nevertheless, there is a lack of studies to support this view. So further RCTs are needed to validate our findings and provide references for clinical treatment.

The estimated glomerular filtration rate (eGFR) is particularly important in the evaluation of renal function (Rebholz et al., 2017). This study indicates that the use of non-steroidal MRAs (finerenone and esaxerenone) in patients with CKD and T2D may lead to decreased eGFR. In contrast, the previous meta-analysis showed that finerenone did not significantly decrease the eGFR in patients with CKD (Fu et al., 2021). We found that there was significant heterogeneity (I^2^ = 86%) among the included studies, and a significant difference was found in changes in eGFR between the finerenone group and the placebo group after excluding one study (*p* = 0.01, I^2^ = 0%) (Fu et al., 2021). Due to the limited number of included studies, they finally reported the former results. Similarly, at first, we did not find a significant difference between the finerenone group and the placebo group in changes in eGFR. Then, we removed one study with a small sample size, and the heterogeneity (I^2^) was reduced from 75.8% to 0%. Meanwhile, the eGFR results were significantly changed (a greater decrease in eGFR was found in the finerenone group than in the placebo group, WMD −2.45%, 95% CI −2.83 to −2.07, *p* < 0.001). However, the pooled effect estimates (finerenone and esaxerenone) remained relatively stable after removing the study with a small sample size. Interestingly, we found that finerenone might have benefits in reducing the incidence of a sustained decrease of 40% in the eGFR from baseline. This finding demonstrates that finerenone does not substantially decrease the eGFR. A recent study revealed that even a 30% increase in serum creatinine did not have an adverse effect on kidney and cardiovascular outcomes with antihypertensive treatment (Collard et al., 2020). Besides, a fall in eGFR is hemodynamically mediated and reversible, because the level plateaued and returned to baseline after cessation of esaxerenone treatment (Ito et al., 2019). Therefore, these reductions in eGFR caused by using non-steroidal MRAs seem to be considered acceptable in clinical settings. Indirect treatment comparisons indicate that finerenone seems to be superior to esaxerenone in alleviating the decline in eGFR. However, the WMD of changes in eGFR from baseline (finerenone vs. esaxerenone) was 2.46 ml/min/1.73 m^2^, which is generally unconsidered in clinical settings. The difference of baseline eGFR may impact on the results, so we removed a study which baseline eGFR lower than 60 ml/min/1.73 m^2^ and found that there was no longer a significant difference between non-steroidal MRAs group and placebo group. Besides, the results of indirect comparisons showed the similar changes between finerenone group and esaxerenone group. The corresponding 95% CI became wider and crossed over one (−1.669 to 11.369, *p* = 0.083). Thus, this further showed that the difference of changes in eGFR from baseline was tiny between finerenone and esaxerenone.

The safety profiles of non-steroidal MRAs should also be considered in clinical treatment. This analysis indicates that all non-steroidal MRAs (finerenone, apararenone, and esaxerenone) do not result in an increased risk of SAEs in patients with CKD and T2D, and no significant difference in the incidence of SAEs was noted among these drugs. The use of finerenone or esaxerenone is associated with a similar higher risk of hyperkalemia, which may result from the treatment mechanism of MRAs. The MRAs increase sodium excretion and decrease potassium excretion in the kidney, leading to a rise in serum potassium levels (Patel et al., 2021). Recent studies have shown that finerenone is associated with a lower risk of hyperkalemia than classic steroidal MRAs (eplerenone or spironolactone) (Yang et al., 2019; Patel et al., 2021). The risk of hyperkalemia in apararenone is unclear owing to a lack of data. These aforementioned evidences indicate finerenone and esaxerenone have receivable and moderate risk of hyperkalemia in patients with CKD and T2D.

This meta-analysis has several limitations. First, the number of studies enrolled in the apararenone group and esaxerenone group was small, and some outcomes were unavailable in the included studies. Second, the number of participants are significantly different in these intervention groups [finerenone (6760), apararenone (253), and esaxerenone (436)]. Third, significant heterogeneity was found in the pooled results of some outcomes, which might result from the effect of the small sample size and different durations of these included RCTs. Fourth, the results of indirect treatment comparisons are insufficiently rigorous due to the small number of enrolled studies. Therefore, head-to-head RCTs are needed to compare the differences of efficacy and safety in these non-steroidal MRAs.

## Conclusion

This meta-analysis indicates that use of non-steroidal MRAs (finerenone, apararenone, and esaxerenone) in patients with CKD and T2D may decreases UACR and SBP without an excess risk of SAEs. Meanwhile, the use of finerenone or esaxerenone in patients with CKD and T2D may lead to an acceptable decrease in eGFR and a moderate increase in the risk of hyperkalemia. In terms of renoprotection, apararenone is likely to be superior to finerenone in decreasing UACR, and esaxerenone and finerenone may have similar effects. Esaxerenone and apararenone may have better antihypertensive effects than finerenone. However, further RCTs that directly compare the efficacy and safety of these drugs in patients with CKD and T2D are needed.

## Abbreviations

ACE, Angiotensin-converting-enzyme; ARB, Angiotensin-receptor blocker; CENTRAL, Cochrane Central Register of Controlled Trials; CI, Confidence interval; CKD, Chronic kidney disease; eGFR, Estimated glomerular filtration rate; ESRD, End-stage renal disease; GLP-1RA, Glucagon-like peptide-1 receptor agonist; MRA, Mineralocorticoid receptor antagonist; MR, Mineralocorticoid receptor; RAS, Renin-angiotensin system; RCT, Randomized controlled trial; RAAS, Renin-angiotensin-aldosterone system; SAE, Serious adverse event; PRISMA, Preferred Reporting Items for Systematic reviews and Meta-Analyses; SBP, Systolic blood pressure; SGLT2, Sodium-glucose cotransporter 2; RR, Risk ratio; T2D, Type 2 diabetes; UACR, Urinary albumin-to-creatinine ratio; WMD, Weighted mean difference.

## References

[B1] Al Dhaybi O. Bakris G. L. (2020). Non-steroidal Mineralocorticoid Antagonists: Prospects for Renoprotection in Diabetic Kidney Disease. Diabetes Obes. Metab. 22 (Suppl. 1), 69–76. 10.1111/dom.13983 32267074

[B2] American Diabetes Association (2020). Microvascular Complications and Foot Care: Standards of Medical Care in Diabetes-2020. Diabetes Care 43 (Suppl. 1), S135–S151. 10.2337/dc20-S011 31862754

[B3] Bärfacker L. Kuhl A. Hillisch A. Grosser R. Figueroa-Pérez S. Heckroth H. (2012). Discovery of BAY 94-8862: A Nonsteroidal Antagonist of the Mineralocorticoid Receptor for the Treatment of Cardiorenal Diseases. ChemMedChem 7 (8), 1385–1403. 10.1002/cmdc.201200081 22791416

[B4] Bakris G. L. Agarwal R. Anker S. D. Pitt B. Ruilope L. M. Rossing P. (2020). Effect of Finerenone on Chronic Kidney Disease Outcomes in Type 2 Diabetes. N. Engl. J. Med. 383 (23), 2219–2229. 10.1056/NEJMoa2025845 33264825

[B5] Bakris G. L. Agarwal R. Chan J. C. Cooper M. E. Gansevoort R. T. Haller H. (2015). Effect of Finerenone on Albuminuria in Patients with Diabetic Nephropathy: A Randomized Clinical Trial. JAMA 314 (9), 884–894. 10.1001/jama.2015.10081 26325557

[B6] Bakris G. Pergola P. E. Delgado B. Genov D. Doliashvili T. Vo N. (2021). Effect of KBP-5074 on Blood Pressure in Advanced Chronic Kidney Disease: Results of the BLOCK-CKD Study. Hypertension 78 (1), 74–81. 10.1161/hypertensionaha.121.17073 33966452 PMC8189259

[B7] Bianchi S. Bigazzi R. Campese V. M. (2006). Long-term Effects of Spironolactone on Proteinuria and Kidney Function in Patients with Chronic Kidney Disease. Kidney Int. 70 (12), 2116–2123. 10.1038/sj.ki.5001854 17035949

[B8] Brandt-Jacobsen N. H. Johansen M. L. Rasmussen J. Forman J. L. Holm M. R. Faber J. (2021). Effect of High-Dose Mineralocorticoid Receptor Antagonist Eplerenone on Urinary Albumin Excretion in Patients with Type 2 Diabetes and High Cardiovascular Risk: Data from the MIRAD Trial. Diabetes Metabol. 47 (4), 101190. 10.1016/j.diabet.2020.08.005 32919068

[B9] Bucher H. C. Guyatt G. H. Griffith L. E. Walter S. D. (1997). The Results of Direct and Indirect Treatment Comparisons in Meta-Analysis of Randomized Controlled Trials. J. Clin. Epidemiol. 50 (6), 683–691. 10.1016/s0895-4356(97)00049-8 9250266

[B10] Collard D. Brouwer T. F. Olde Engberink R. H. G. Zwinderman A. H. Vogt L. van den Born B. H. (2020). Initial Estimated Glomerular Filtration Rate Decline and Long-Term Renal Function during Intensive Antihypertensive Therapy: A Post Hoc Analysis of the SPRINT and ACCORD-BP Randomized Controlled Trials. Hypertension 75 (5), 1205–1212. 10.1161/HYPERTENSIONAHA.119.14659 32223381 PMC7176351

[B11] Cumpston M. Li T. Page M. J. Chandler J. Welch V. A. Higgins J. P. (2019). Updated Guidance for Trusted Systematic Reviews: A New Edition of the Cochrane Handbook for Systematic Reviews of Interventions. Cochrane Database Syst. Rev. 10, ED000142. 10.1002/14651858.ED000142 31643080 PMC10284251

[B12] Delanaye P. Scheen A. J. (2019). Preventing and Treating Kidney Disease in Patients with Type 2 Diabetes. Expert Opin. Pharmacother. 20 (3), 277–294. 10.1080/14656566.2018.1551362 30462565

[B13] El Mokadem M. Abd El Hady Y. Aziz A. (2020). A Prospective Single-Blind Randomized Trial of Ramipril, Eplerenone and Their Combination in Type 2 Diabetic Nephropathy. Cardiorenal Med. 10 (6), 392–401. 10.1159/000508670 32998143

[B14] Epstein M. Williams G. H. Weinberger M. Lewin A. Krause S. Mukherjee R. (2006). Selective Aldosterone Blockade with Eplerenone Reduces Albuminuria in Patients with Type 2 Diabetes. Clin. J. Am. Soc. Nephrol. 1 (5), 940–951. 10.2215/CJN.00240106 17699311

[B15] European Union Clinical Trials Register (2016). A Study to Evaluate Pharmacodynamics, Safety, Tolerability and Pharmacokinetics of MT-3995 in Type II Diabetic Nephropathy Subjects with Albuminuria and Moderately Decreased GFR. Available at: https://www.clinicaltrialsregister.eu/ctr-search/trial/2012-002481-12/results (Accessed December 6, 2021).

[B16] Fu Z. Geng X. Chi K. Song C. Wu D. Liu C. (2021). Efficacy and Safety of Finerenone in Patients with Chronic Kidney Disease: A Systematic Review with Meta-Analysis and Trial Sequential Analysis. Ann. Palliat. Med. 10 (7), 7428–7439. 10.21037/apm-21-763 34353035

[B17] Gansevoort R. T. Verhave J. C. Hillege H. L. Burgerhof J. G. Bakker S. J. de Zeeuw D. (2005). The Validity of Screening Based on Spot Morning Urine Samples to Detect Subjects with Microalbuminuria in the General Population. Kidney Int. Suppl. (94), S28–S35. 10.1111/j.1523-1755.2005.09408.x 15752236

[B18] Ingelfinger J. R. Rosen C. J. (2020). Finerenone - Halting Relative Hyperaldosteronism in Chronic Kidney Disease. N. Engl. J. Med. 383 (23), 2285–2286. 10.1056/NEJMe2031382 33095527

[B19] Ito S. Kashihara N. Shikata K. Nangaku M. Wada T. Okuda Y. (2020). Esaxerenone (CS-3150) in Patients with Type 2 Diabetes and Microalbuminuria (ESAX-DN): Phase 3 Randomized Controlled Clinical Trial. Clin. J. Am. Soc. Nephrol. 15 (12), 1715–1727. 10.2215/CJN.06870520 33239409 PMC7769030

[B20] Ito S. Shikata K. Nangaku M. Okuda Y. Sawanobori T. (2019). Efficacy and Safety of Esaxerenone (CS-3150) for the Treatment of Type 2 Diabetes with Microalbuminuria: A Randomized, Double-Blind, Placebo-Controlled, Phase II Trial. Clin. J. Am. Soc. Nephrol. 14 (8), 1161–1172. 10.2215/CJN.14751218 31248950 PMC6682830

[B21] Kang Y. S. Cha D. R. (2009). Aldosterone and Diabetic Kidney Disease. Curr. Diabetes Rep. 9 (6), 453–459. 10.1007/s11892-009-0074-x 19954691

[B22] Katayama S. Yamada D. Nakayama M. Yamada T. Myoishi M. Kato M. (2017). A Randomized Controlled Study of Finerenone versus Placebo in Japanese Patients with Type 2 Diabetes Mellitus and Diabetic Nephropathy. J. Diabetes Complicat. 31 (4), 758–765. 10.1016/j.jdiacomp.2016.11.021 28025025

[B23] Kolkhof P. Delbeck M. Kretschmer A. Steinke W. Hartmann E. Bärfacker L. (2014). Finerenone, a Novel Selective Nonsteroidal Mineralocorticoid Receptor Antagonist Protects from Rat Cardiorenal Injury. J. Cardiovasc. Pharmacol. 64 (1), 69–78. 10.1097/FJC.0000000000000091 24621652

[B24] Lee J. Y. Park J. T. Joo Y. S. Lee C. Yun H. R. Yoo T. H. (2021). Association of Blood Pressure with the Progression of CKD: Findings from KNOW-CKD Study. Am. J. Kidney Dis. 78 (2), 236–245. 10.1053/j.ajkd.2020.12.013 33444666

[B25] Li H. Lu W. Wang A. Jiang H. Lyu J. (2021). Changing Epidemiology of Chronic Kidney Disease as a Result of Type 2 Diabetes Mellitus from 1990 to 2017: Estimates from Global Burden of Disease 2017. J. Diabetes Investig. 12 (3), 346–356. 10.1111/jdi.13355 PMC7926234 32654341

[B26] Lin Y. C. Chang Y. H. Yang S. Y. Wu K. D. Chu T. S. (2018). Update of Pathophysiology and Management of Diabetic Kidney Disease. J. Formos. Med. Assoc. 117 (8), 662–675. 10.1016/j.jfma.2018.02.007 29486908

[B27] Moher D. Liberati A. Tetzlaff J. Altman D. G. PRISMA Group (2009). Preferred Reporting Items for Systematic Reviews and Meta-Analyses: the PRISMA Statement. BMJ 339, b2535. 10.1136/bmj.b2535 19622551 PMC2714657

[B28] Norris K. C. Smoyer K. E. Rolland C. Van der Vaart J. Grubb E. B. (2018). Albuminuria, Serum Creatinine, and Estimated Glomerular Filtration Rate as Predictors of Cardio-Renal Outcomes in Patients with Type 2 Diabetes Mellitus and Kidney Disease: A Systematic Literature Review. BMC Nephrol. 19 (1), 36. 10.1186/s12882-018-0821-9 29426298 PMC5807748

[B29] Patel V. Joharapurkar A. Jain M. (2021). Role of Mineralocorticoid Receptor Antagonists in Kidney Diseases. Drug Dev. Res. 82 (3), 341–363. 10.1002/ddr.21760 33179798

[B30] Perkovic V. Jardine M. J. Neal B. Bompoint S. Heerspink H. J. L. Charytan D. M. (2019). Canagliflozin and Renal Outcomes in Type 2 Diabetes and Nephropathy. N. Engl. J. Med. 380 (24), 2295–2306. 10.1056/NEJMoa1811744 30990260

[B31] Pitt B. Filippatos G. Agarwal R. Anker S. D. Bakris G. L. Rossing P. (2021). Cardiovascular Events with Finerenone in Kidney Disease and Type 2 Diabetes. N. Engl. J. Med. 385 (24), 2252–2263. 10.1056/NEJMoa2110956 34449181

[B32] Pitt B. Kober L. Ponikowski P. Gheorghiade M. Filippatos G. Krum H. (2013). Safety and Tolerability of the Novel Non-steroidal Mineralocorticoid Receptor Antagonist BAY 94-8862 in Patients with Chronic Heart Failure and Mild or Moderate Chronic Kidney Disease: A Randomized, Double-Blind Trial. Eur. Heart J. 34 (31), 2453–2463. 10.1093/eurheartj/eht187 23713082 PMC3743070

[B33] Rebholz C. M. Inker L. A. Chen Y. Liang M. Foster M. C. Eckfeldt J. H. (2017). Risk of ESRD and Mortality Associated with Change in Filtration Markers. Am. J. Kidney Dis. 70 (4), 551–560. 10.1053/j.ajkd.2017.04.025 28648303 PMC5610931

[B34] Sun L. J. Sun Y. N. Shan J. P. Jiang G. R. (2017). Effects of Mineralocorticoid Receptor Antagonists on the Progression of Diabetic Nephropathy. J. Diabetes Investig. 8 (4), 609–618. 10.1111/jdi.12629 PMC5497036 28107779

[B35] Takahashi M. Ubukata O. Homma T. Asoh Y. Honzumi M. Hayashi N. (2020). Crystal Structure of the Mineralocorticoid Receptor Ligand-Binding Domain in Complex with a Potent and Selective Nonsteroidal Blocker, Esaxerenone (CS-3150). FEBS Lett. 594 (10), 1615–1623. 10.1002/1873-3468.13746 31991486

[B36] Thomas M. C. Brownlee M. Susztak K. Sharma K. Jandeleit-Dahm K. A. Zoungas S. (2015). Diabetic Kidney Disease. Nat. Rev. Dis. Prim. 1, 15018. 10.1038/nrdp.2015.18 27188921 PMC7724636

[B37] Wada T. Inagaki M. Yoshinari T. Terata R. Totsuka N. Gotou M. (2021). Apararenone in Patients with Diabetic Nephropathy: Results of a Randomized, Double-Blind, Placebo-Controlled Phase 2 Dose-Response Study and Open-Label Extension Study. Clin. Exp. Nephrol. 25 (2), 120–130. 10.1007/s10157-020-01963-z 32974732 PMC7880964

[B38] Weir M. R. Lakkis J. I. Jaar B. Rocco M. V. Choi M. J. Kramer H. J. (2018). Use of Renin-Angiotensin System Blockade in Advanced CKD: An NKF-KDOQI Controversies Report. Am. J. Kidney Dis. 72 (6), 873–884. 10.1053/j.ajkd.2018.06.010 30201547

[B39] Yang P. Shen W. Chen X. Zhu D. Xu X. Wu T. (2019). Comparative Efficacy and Safety of Mineralocorticoid Receptor Antagonists in Heart Failure: A Network Meta-Analysis of Randomized Controlled Trials. Heart Fail Rev. 24 (5), 637–646. 10.1007/s10741-019-09790-5 31030322

