# Supplementary material for: Efficacy and Safety of Non-Steroidal Mineralocorticoid Receptor Antagonists in Patients With Chronic Kidney Disease and Type 2 Diabetes: A Systematic Review Incorporating an Indirect Comparisons Meta-Analysis
Source: Front Pharmacol. 2022 Jun 16;13:896947. doi: 10.3389/fphar.2022.896947 (PMC9243561; doi:10.3389/fphar.2022.896947)
Supplement: Supplementary file 1 [file Table1.DOCX]

**Search strategy for the PubMed**

①((((((Finerenone[Title/Abstract]) OR (BAY94-8862[Title/Abstract])) OR (kerendia[Title/Abstract])) OR (MT-3995[Title/Abstract])) OR (Apararenone[Title/Abstract])) OR (esaxerenone[Title/Abstract])) OR (1-(2-hydroxyethyl)-4-methyl-N-(4-(methylsulfonyl)phenyl)-5-(2-(trifluoromethyl)phenyl)-1H-pyrrole-3-carboxamide[Title/Abstract]))

②(((((((((((((((((((Mineralocorticoid Receptor Antagonists[Title/Abstract]) OR (Antagonists, Mineralocorticoid Receptor[Title/Abstract])) OR (Receptor Antagonists, Mineralocorticoid[Title/Abstract])) OR (Mineralocorticoid Antagonists[Title/Abstract])) OR (Antagonists, Mineralocorticoid[Title/Abstract])) OR (Aldosterone Receptor Antagonist[Title/Abstract])) OR (Antagonist, Aldosterone Receptor[Title/Abstract])) OR (Receptor Antagonist, Aldosterone[Title/Abstract])) OR (Mineralocorticoid Antagonist[Title/Abstract])) OR (Antagonist, Mineralocorticoid[Title/Abstract])) OR (Mineralocorticoid Receptor Antagonist[Title/Abstract])) OR (Antagonist, Mineralocorticoid Receptor[Title/Abstract])) OR (Receptor Antagonist, Mineralocorticoid[Title/Abstract])) OR (Aldosterone Receptor Antagonists[Title/Abstract])) OR (Antagonists, Aldosterone Receptor[Title/Abstract])) OR (Receptor Antagonists, Aldosterone[Title/Abstract])) OR (Aldosterone Antagonists[Title/Abstract])) OR (Antagonists, Aldosterone[Title/Abstract])) OR (Aldosterone Antagonist[Title/Abstract])) OR (Antagonist, Aldosterone[Title/Abstract])

③(((((((((((((("Diabetic Nephropathies"[Mesh]) OR (Nephropathies, Diabetic[Title/Abstract])) OR (Nephropathy, Diabetic[Title/Abstract])) OR (Nephropathy, Diabetic[Title/Abstract])) OR (Diabetic Nephropathy[Title/Abstract])) OR (Diabetic Kidney Diseases[Title/Abstract])) OR (Kidney Disease, Diabetic[Title/Abstract])) OR (Kidney Diseases, Diabetic[Title/Abstract])) OR (Diabetic Glomerulosclerosis[Title/Abstract])) OR (Glomerulosclerosis, Diabetic[Title/Abstract])) OR (Intracapillary Glomerulosclerosis[Title/Abstract])) OR (Nodular Glomerulosclerosis[Title/Abstract])) OR (Kimmelstiel-Wilson Syndrome[Title/Abstract])) OR (Syndrome, Kimmelstiel-Wilson[Title/Abstract])) OR (Kimmelstiel-Wilson Disease[Title/Abstract])

④Diabetes Mellitus[Title/Abstract])

⑤(((Disease, Kidney[Title/Abstract]) OR (Diseases, Kidney[Title/Abstract])) OR (Kidney Disease[Title/Abstract])) OR (nephropathy[Title/Abstract])

⑥((((((randomized controlled trial[Publication Type]) OR controlled clinical trial[Publication Type]) OR randomized[Title/Abstract]) OR placebo[Title/Abstract]) OR randomly[Title/Abstract]) OR trial[Title/Abstract])

⑦：① or ②

⑧：③ or ④ or ⑤

⑨：⑦ and ⑧ and ⑥
