# Supplementary figures and images for: Efficacy and Safety of Non-Steroidal Mineralocorticoid Receptor Antagonists in Patients With Chronic Kidney Disease and Type 2 Diabetes: A Systematic Review Incorporating an Indirect Comparisons Meta-Analysis
Source: Front Pharmacol. 2022 Jun 16;13:896947. doi: 10.3389/fphar.2022.896947 (PMC9243561; doi:10.3389/fphar.2022.896947)

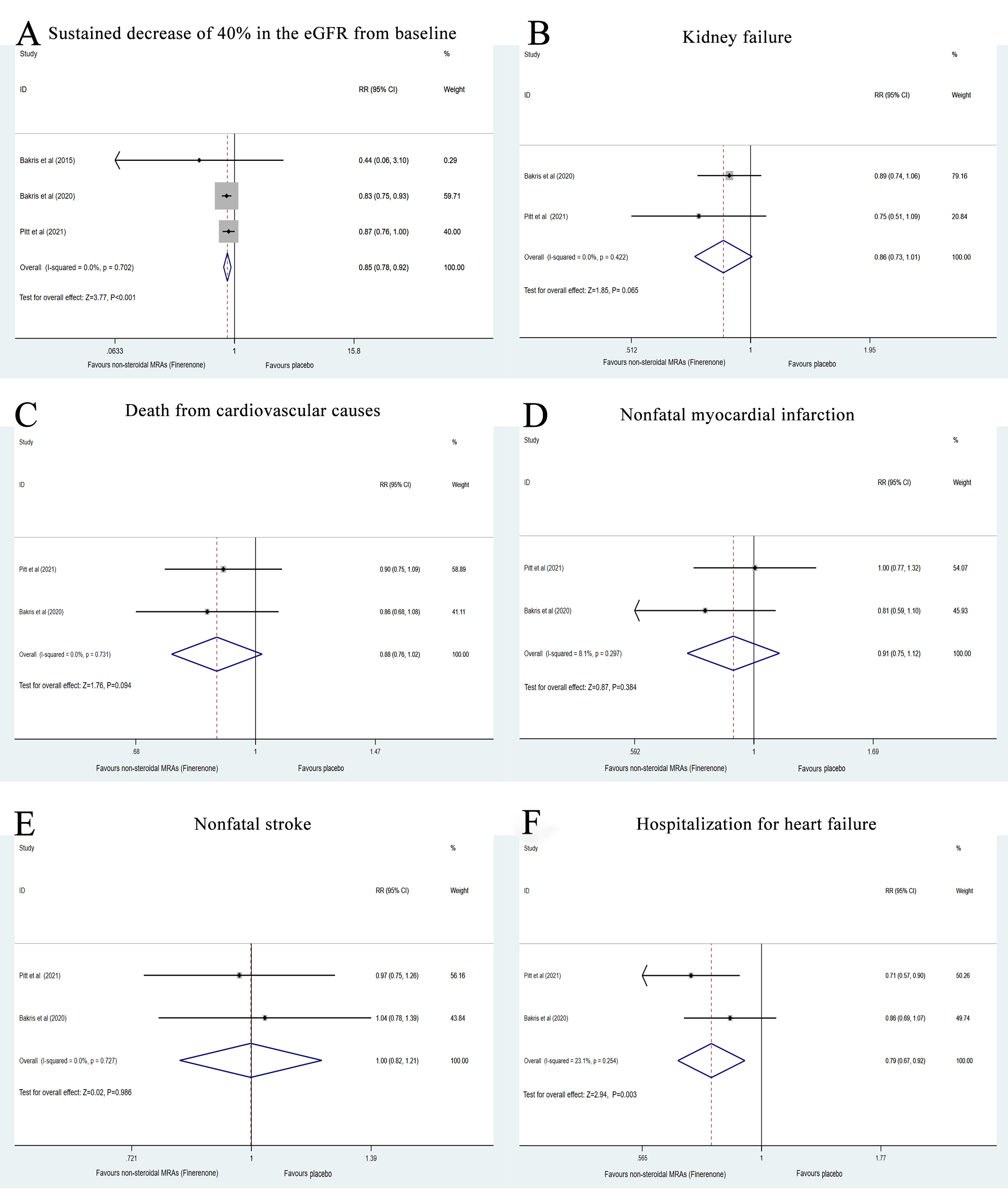

Supplement: Supplementary file 2 [file Image2.TIF]

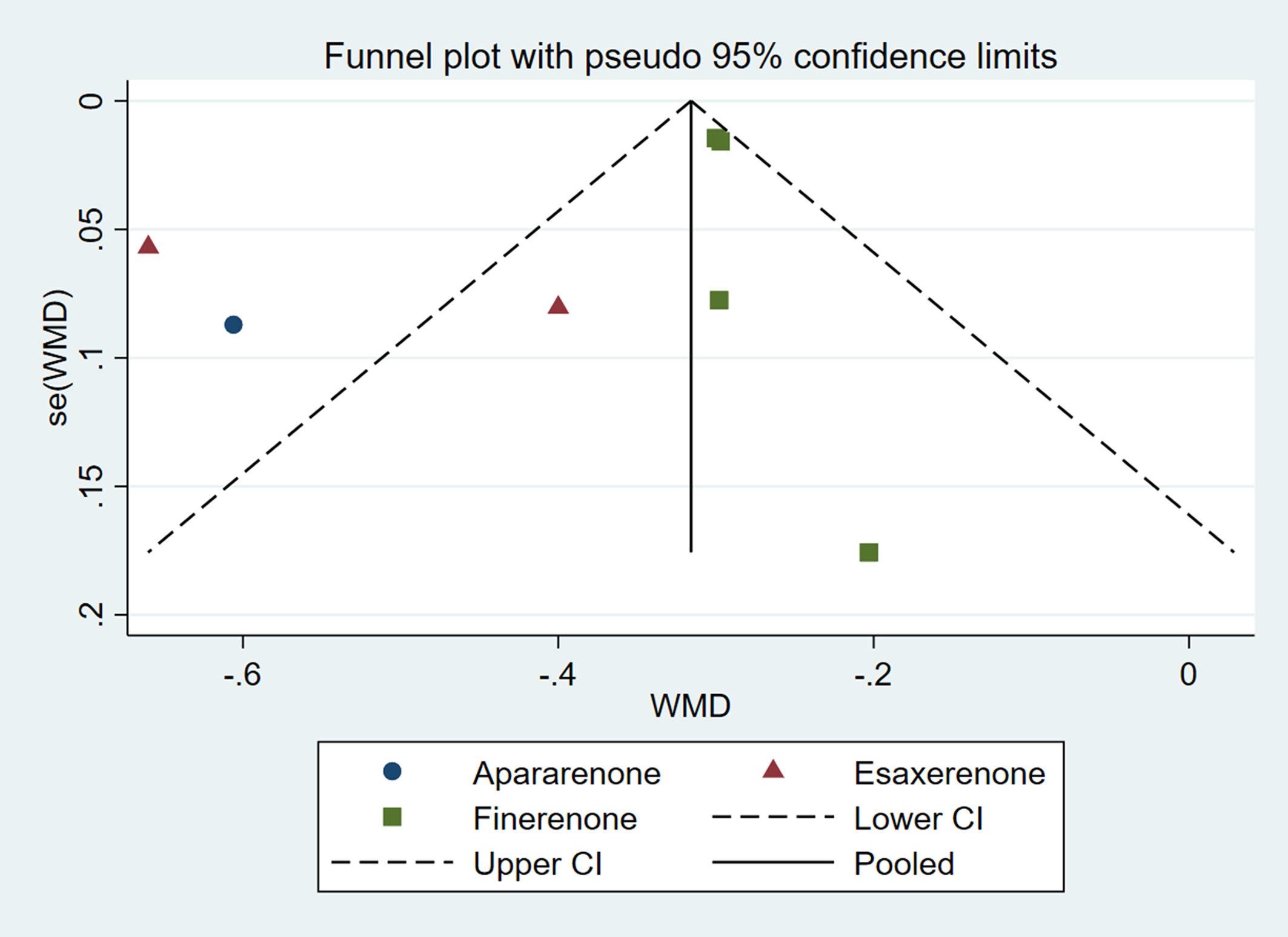

Supplement: Supplementary file 3 [file Image1.TIF]
