# Supplementary material for: Efficacy and Safety of Non-Steroidal Mineralocorticoid Receptor Antagonists in Patients With Chronic Kidney Disease and Type 2 Diabetes: A Systematic Review Incorporating an Indirect Comparisons Meta-Analysis
Source: Front Pharmacol. 2022 Jun 16;13:896947. doi: 10.3389/fphar.2022.896947 (PMC9243561; doi:10.3389/fphar.2022.896947)
Supplement: Supplementary file 4 [file Table2.DOCX]

| Supplementary Table S2 Direct and indirect comparisons after matching baseline characteristics | | | | | | | | |
| --- | --- | --- | --- | --- | --- | --- | --- | --- |
|  | Direct comparisons (vs. placebo) | | | |  | Indirect comparisons | | |
| Outcomes | Finerenone | Apararenone | Esaxerenone | Pooled non-steroidal MRAs |  | Finerenone vs. Apararenone | Finerenone vs. Esaxerenone | Apararenone vs. Esaxerenone |
| Changes in UACR from baseline^*^ | -0.28 (-0.42 to -0.14), P<0.001 | -0.61 (-0.78 to -0.44), P<0.001 | -0.54 (-0.79 to -0.28), P<0.001 | -0.46 (-0.63 to -0.29), P<0.001 |  | 0.33 (0.11 to 0.55), P=0.013 | 0.26 (-0.031 to 0.551), P=0.919 | -0.07 (-0.376 to 0.236), P=0.979 |
| Changes in eGFR from baseline^#^ | -0.06 (-5.22 to 5.09), P=0.981 | NA | -4.91 (-6.64 to -3.19), P<0.001 | -2.64 (-5.65 to 0.36), P=0.084 |  | NA | 4.85 (-1.669 to 11.369), P=0.083 | NA |
| Incidence of hyperkalemia^$^ | 2.04 (1.72 to 2.40), P<0.001 | NA | 4.45 (1.99 to 9.97), P<0.001 | 2.13 (1.81 to 2.51), P<0.001 |  | NA | 0.458 (0.201 to 1.044), P=0.166 | NA |
| ^*^ Studies were included according to the UACR at baseline (≤300 mg/g), and data was presented as weighted mean difference (95% CI); ^#^ Studies were included according to the eGFR at baseline (≥60 mL/min/1.73 m^2^), and data was presented as weighted mean difference (95% CI); ^$^ Studies were included according to the eGFR at baseline (≥60 mL/min/1.73 m^2^), and data was presented as risk ratio (95% CI); UACR, urinary albumin-creatinine ratio; eGFR, estimated glomerular filtration rate; NA, not available | | | | | | | | |
